# Alexithymia in patients with Parkinson’s disease treated with DBS of the subthalamic nucleus: a case-control study

**DOI:** 10.3389/fpsyg.2014.01168

**Published:** 2014-10-13

**Authors:** Lorys Castelli, Debora Tonello, Laura Rizzi, Maurizio Zibetti, Michele Lanotte, Leonardo Lopiano

**Affiliations:** ^1^Department of Psychology, University of TurinTurin, Italy; ^2^Department of Neuroscience, University of TurinTurin, Italy

**Keywords:** alexithymia, mood, emotional dysregulation, PD patients, STN-DBS surgery

## Abstract

**Objectives:** To evaluate the effect of deep brain stimulation of the subthalamic nucleus (STN-DBS) on alexithymia, a deficit in affective regulation, comparing patients with Parkinson’s disease (PD) submitted to STN-DBS (DBS group) to PD patients not yet treated with STN-DBS (pre-DBS group) and to healthy participants (C group).

**Methods**: We recruited 27 consecutive STN-DBS PD patients, 38 consecutive pre-DBS patients and 27 healthy participants. Patients were assessed for alexithymia (Toronto Alexithymia Scale), depression, [beck depression inventory (BDI)], and cognitive functions (reasoning, memory, attentional, and executive tests).

**Results:** The DBS patients performed worse than the pre-DBS patients in the corsi’s block-tapping test, in the phonemic fluency task and in the Frontal Assessment Battery. Around 30% of DBS (29.6%) and pre-DBS (31.6%) patients resulted alexithymic, compared with 14.8% in the C group. The results pointed out significantly higher alexithymia scores in both the DBS and pre-DBS groups compared with the C group, while no difference emerged between the DBS and pre-DBS groups. Pre-DBS group showed a significantly higher BDI score than the C group, while DBS group did not.

**Conclusion:** Although the results suggest that STN-DBS does not affect alexithymia, both the DBS and pre-DBS patients reported higher prevalence (about 30%) of alexithymia than did healthy subjects (14.8%).

## INTRODUCTION

Parkinson’s disease (PD) is a common progressive, disabling neurodegenerative disorder with onset of motor and non-motor features (Müller, 2012), namely with substantial physical, psychological, and social implication (Worth, 2013).

Deep brain stimulation of the subthalamic nucleus (STN-DBS) is a clinically established procedure for the treatment of motor symptoms in patients with advanced PD (Witt et al., 2008; Gervais-Bernard et al., 2009; Odekerken et al., 2013). Although several studies have demonstrated its efficacy on motor symptoms, its impact on cognitive and behavioral/psychological aspects is still controversial and debated (Parsons et al., 2006; Castelli et al., 2008; Witt et al., 2008; Gervais-Bernard et al., 2009). Some studies have concluded that STN-DBS is safe from a cognitive standpoint since it does not lead to general cognitive deterioration (Castelli et al., 2006; Parsons et al., 2006), others have evidenced a negative impact on specific cognitive domains such as memory, attention, verbal learning, fluency tasks, and executive functions (Witt et al., 2008; Castelli et al., 2010).

Neuropsychiatric complications such as aggressive and psychotic episodes, apathy, hypomania/mania, depressive episodes, and affect dysregulation can occasionally, and often transitively, occur in the post-operative period (Castelli et al., 2006; Witt et al., 2008, 2012; Strutt et al., 2012). To date, there is no single explanation for these neuropsychiatric complications: they could depend on both the indirect influence of STN stimulation on the limbic system, including the influence of electrode placement (Strutt et al., 2012), and on the post-operative reduction of dopaminergic therapy.

A general consensus has not yet been reached on mood changes after DBS of STN: most studies have reported either no change or an improvement in depression after surgery, while others have reported a worsening of mood state, including suicidal ideation and attempted completed suicide (Gervais-Bernard et al., 2009; Strutt et al., 2012; Weintraub et al., 2013), particularly in patients with a previous history of psychiatric disorder (Lilleeng and Dietrichs, 2008).

One study does not support direct association between DBS surgery and increased risk for suicidal ideation and behaviors (Weintraub et al., 2013), while another one highlights that postoperative depression remained a significant factor associated with attempted and completed suicide (Voon et al., 2008). Given the lack of consensus, the limited number of studies and the great psycho-social impact of post-operative neuropsychiatric and psychological disturbances, these issues warrant further investigation.

On this basis, the present study aimed at investigating alexithymia, a deficit in affect regulation that has received little attention with regard to PD patients, despite its definition in a recent study as “a non-motor symptom of PD” (Assogna et al., 2012). Alexithymia is a personality dimension characterized by an impairment in identifying and describing feelings, and an impairment in distinguishing between feelings and bodily sensations due to emotional arousal, compromised symbolization, and an externally oriented cognitive style, i.e., a tendency to selectively focus on everything is outward, factual and concrete and to report events and action without affective involvement (Bagby et al., 1994). Alexithymia can therefore be defined as an inability to recognize and to communicate emotions. Since previous studies have shown a high prevalence of this disturbance in PD patients (Assogna et al., 2012) and since affect dysregulation symptoms have been shown to have a relevant impact on PD patients (Costa et al., 2010; Assogna et al., 2012), especially in the ones treated with DBS surgery (Strutt et al., 2012), further study on this topic are necessary.

To date, no study has addressed the possible impact of STN-DBS on alexithymia, even though the few studies assessing alexithymia in PD patients (not treated with STN-DBS) have shown a high prevalence – about 20% – of this disturbance (Costa et al., 2010; Assogna et al., 2012).

The main objective of this study is to investigate the effects of STN-DBS on alexithymia by means of a case-control study comparing a DBS PD group to a pre-DBS PD group and to a control group of healthy participants. Secondarily, patients were also assessed for depressive symptoms and cognitive functions. Cognitive functions were assessed since deficits in specific cognitive domains, especially executive functions, could arise after STN-DBS.

## MATERIALS AND METHODS

### PATIENTS

Three groups of subjects were involved in the case-control design: 27 consecutive PD patients bilaterally implanted for DBS of STN (DBS group), 38 consecutive PD patients under dopaminergic therapy (PRE-DBS group), and 27 healthy control subjects (C group). All the participants gave their written informed consent to the study after approval by the Ethics Committee of the University of Turin Medical School. Confidentiality and the right to withdraw from the study were guaranteed to all the participants.

The patients of the DBS group successfully underwent DBS of STN and were hospitalized at San Giovanni Battista Hospital to adjust antiparkinsonian medication and electrical stimulator parameters (1 year follow-up). The main inclusion criteria for the surgical STN-DBS treatment were: diagnosis of idiopathic PD, severe motor fluctuations, drug-related dyskinesias, age under 70. The main exclusion criteria were: marked atrophy or focal abnormalities on brain MRI, dementia, or relevant cognitive decline, severe depression, and/or psychiatric disorders at the time of the pre-surgical evaluation.

The patients of the pre-DBS group had not yet been surgically treated, but satisfied the same inclusion criteria as the DBS group; they were judged suitable for the surgical treatment and were on the waiting list for STN-DBS surgery.

The two PD groups were matched for sex, age, years of education, duration, and severity of illness (see **Table 1**). To evaluate the severity of motor symptoms in order to match the two PD groups, we used Unified Parkinson’s Disease Rating Scale (UPDRS) -part III – motor scores (Members of the UPDRS development committee et al., 1987): the DBS group was evaluated in medication-off (MED-OFF)/stimulation-off (STIM-OFF) condition (after overnight withdrawal of antiparkinsonian medication and with stimulation switched off for at least 1 h), and the Pre-DBS group in medication-off condition (MED-OFF; after overnight withdrawal of antiparkinsonian medication).

**Table 1:** Clinical characteristics and neuropsychological data of the DBS, pre-DBS, and C groups.

	DBS group	Pre-DBS group	CS group	*F*(df)	*P*
***Clinical characteristics***
Number of subjects	27	38	27	–	–
Sex (male/female)	16/11	23/15	15/12	0.2(2)^a^	–
Age	61.4(8.1)	59(7.4)	60.1(10.7)	0.6(2;89)	0.5
Years of education	9.5(3.6)	9(3.9)	11.2(3.3)	2.9(2;89)	0.06

	**DBS group**	**Pre-DBS group**	**–**	***T*(df)**	**–**

Duration of the dopaminergic treatment (years)	14.2(4.2)	12.7(4.8)	–	–1.5(63)	–
Severity of the disease (UPDRS section III, off condition)	47.3(8.8)	44.3(6.3)	–	–1.6 (63)	–
LEDD (levodopa equivalent daily dosage)	729.0(305.3)	980.1(353.1)	–	3.0 (63)	*0.004*
***Neuropsychological data***

	**DBS group**	**pre-DBS group**	**–**	***T*(df)**	***P***

MMSE	27.9(1.5)	28.5(1.5)	–	1.5(63)	0.1
Corsi’s Block-Tapping Test	5(0.5)	5.5(0.9)	–	2.6(63)	*0.007*
Raven Color Matrices	26.6(4.3)	27.7(5)	–	0.9(63)	0.4
Bisyllabic Word Repetition Test	4.1(08)	4.5(0.9)	–	1.9(63)	0.01
Paired-Associate Learning	10.3(2.8)	11.1(2.7)	–	1.2(63)	0.2
Trail mMaking Test Part B	261.9(165)	198.2(161.5)	–	–1.5(63)	0.1
Nelson Modified Card Sorting Test
Categories	5.6(0.7)	5.8(0.5)	–	1(63)	0.3
Errors	6(5.1)	4.4(3.5)	–	–1.5(63)	0.1
Perseverations	2.6(2.2)	2.3(2.8)	–	–0.4(63)	0.7
Phonemic verbal fluency	24.9(9.7)	33(10.1)	–	3.3(63)	0.002
Category verbal fluency	19(4.3)	20.9(4.5)	–	1.7(63)	0.1
FAB	14.7(1.6)	16.1(1.5)	–	3.5(63)	*0.01*

*Mean (SD) and univariate ANOVAs (*F*)/*t*-tests (*T*) are shown. ^a^chi square.*

After surgery, the DBS group obtained a significant amelioration in UPDRS III scores; motor symptoms decreased by 66.2% (pre-operative MED-OFF condition vs. post-operative STIM-ON/MED-OFF conditions). Moreover, surgery allowed DBS PD patients to reduce their drug intake: comparing pre- (mean: 1045.5 mg; SD: 423.2 mg) and post-surgery levodopa equivalent daily dose (mean: 729.0 mg; SD: 305.3 mg), the DBS group decreased their drug consumption by 30.3% [*t*(26) = 3.9; *p* = 0.001].

The subjects of the C group were matched for age and years of education to PD patients (DBS and pre-DBS groups). The exclusion criteria for this group were: cognitive deficits (MMSE score ≤ 24), the presence of severe systemic or metabolic disease, intake of psychotropic medication, a history of psychiatric or neurological illness, or substance abuse.

All the PD patients (DBS and pre-DBS groups) and subjects of the C group underwent mood and alexithymia evaluation, with the PD patients also undergoing a cognitive assessment. These psychological and neuropsychological evaluations were made in the MED-ON/STIM-ON condition for the DBS group, and in MED-ON for the pre-DBS group.

### COGNITIVE ASSESSMENT

The DBS and pre-DBS patients were given a standardized neuropsychological test battery in order to assess reasoning, memory, and attentional executive functions (Castelli et al., 2010). Visuospatial reasoning was evaluated by means of the Raven Color Matrices (PM 47; Raven, 1962); verbal, and spatial short-term memory was assessed by means of the bisyllabic word repetition test (BWR; Spinnler and Tognoni, 1987) and corsi’s block-tapping test (Spinnler and Tognoni, 1987), respectively. The assessment of verbal learning was achieved by means of the paired-associate learning (PAL; Wechsler, 1945), a Wechsler Memory Scale subtest. Frontal lobe executive functions, including the development of abstract concepts and the shift of attention and motor sets, were assessed by means of the Trail Making Test Part B (Reitan, 1958) and the Nelson Modified Card Sorting Test (MCST; Nelson, 1976), a modified version of the Wisconsin Card Sorting Test. In addition, patients were given phonemic (Benton, 1968) and category (Spinnler and Tognoni, 1987) verbal fluency tasks and a specific battery for frontal functions, the Frontal Assessment Battery (FAB; Dubois et al., 2000).

### ALEXITHYMIA AND MOOD ASSESSMENT

Alexithymia was assessed by means of the Toronto Alexithymia Scale (TAS-20; Bagby et al., 1994). TAS-20 is a validated self-report 20-item questionnaire with a five-point Likert response scale. It is composed of three subscales that investigate three factors: difficulty identifying feelings (F1; e.g., “I am often confused about what emotion I am feeling”); difficulty describing feelings (F2; e.g., “I am often confused about what emotion I am feeling”), difficulty focusing on inner affective experience (F3; e.g., “I prefer talking to people about their daily activities rather than their feelings”).

The total score ranges from 20 to 100 and allows subjects to be categorized as “Non-alexithymic” (score from 20 to 50), “Borderline” (score from 51 to 60), and “Alexithymic” (score ≥ 61; Castelli et al., 2010).

Mood was evaluated by means of the Beck Depression Inventory (BDI; Beck and Streer, 1987). The BDI, considered a reliable instrument for the evaluation of depression severity in PD, is a 21-item self-rated questionnaire with a four-point Likert response scale (0–3). The total score ranges from 0 (absence of depression) to 63 (severe depression).

### STATISTICAL ANALYSIS

Univariate ANOVAs for unpaired samples were run to compare the test scores of the DBS, pre-DBS, and C groups; Bonferroni analysis was used for *post hoc* two-by-two comparisons.

To compare the DBS and pre-DBS groups, we used a *t*-test for independent samples. χ^2^ analysis was used to compare the different prevalence of subjects among groups, i.e., number of alexithymic subjects in the three groups. Finally, correlations between variables were analyzed through Pearson correlation analysis.

## RESULTS

### NEUROPSYCHOLOGICAL RESULTS: THE DBS GROUP VS PRE-DBS GROUP

The neuropsychological test scores are shown in **Table 1**. The DBS and pre-DBS groups evidenced statistically significant differences in spatial short-term memory (CPT; *p* = 0.007) and in some executive tests, i.e., the phonemic fluency task (*p* = 0.002) and the FAB (*p* = 0.01). In all three tests, the DBS patients performed worse than the pre-DBS patients.

### ALEXITHYMIA AND MOOD

The TAS and BDI scores of the three groups are given in **Table 2**. Univariate ANOVAs showed significant differences between the three groups in the TAS total score (*p* < 0.0001) and F1 scores (*p* < 0.05), but not in the F2 and F3 subscales. With regard to the TAS total score, as well as to the F1 subscale, *post hoc* analysis underlined significantly higher scores both in the DBS and pre-DBS groups compared with the C group, while no difference was found between the DBS and pre-DBS groups.

**Table 2:** Comparison of alexithymia and mood (BDI) between the DBS, pre-DBS, and C groups.

	DBS group	Pre-DBS group	CS group	*F*(df)	*P*	*Post hoc*
**Alexithymia**
TAStot	54(10.1)	53.7(10.9)	46.1(11.2)	5(2;89)	*0.009*	DBS > CS^b^ pre-DBS > CS^b^
F1	18.7(6)	18.4(6.6)	14.3(5.6)	4.5(2;89)	*0.014*	DBS > CS^b^ pre-DBS > CS^b^
F2	13.6(4.2)	14(3.6)	12.4(4.6)	1.4(2;89)	–	
F3	21.7(3.9)	21.2(4.7)	19.4(4.9)	2(2;89)	–	
**Mood**
BDI	10.3(6.1)	14.1(8.1)	9.5(6.5)	4(2;89)	*0.021*	pre-DBS > CS^b^

*Means (SD), univariate ANOVAs, and Bonferroni *post hoc* results are shown. ^b^*p* < 0.05.*

χ^2^ analysis confirmed a significantly different percentage of alexithymic subjects in the three groups (χ^2^ = 9.54; *p* < 0.05). Specifically, in the DBS and pre-DBS groups, 29.6% (8/27) and 31.6% (12/38), respectively, of the subjects resulted alexithymic, compared with 14.8% (4/27) in the C group. As far as subclinical disturbance is concerned (borderline score), 40.7% (11/27), 34.2% (13/38), and 18.5% (5/27) of participants resulted borderline in the DBS, pre-DBS, and CT groups, respectively.

Concerning mood, the three groups had significantly different BDI scores (*p* < 0.05). *Post hoc* analysis highlighted statistically significant differences between the pre-DBS and C groups (*p* < 0.05) while, even if BDI scores of DBS patients are lower than C group ones, this difference was not statistically significant. These data and results are given in **Table 2**.

### CORRELATIONS BETWEEN VARIABLES IN THE PRE-DBS AND DBS GROUP

No significant correlation was found between TAS-20 total score and severity of the disease (UPDRS -part III in the OFF condition) both for the pre-DBS and DBS group.

Also no significant correlation was found between TAS-20 total and subscales scores and neuropsychological test scores, both for the pre-DBS and DBS group.

As far as depression is concerned, correlation analysis showed a significant positive correlation between TAS-20 and BDI scores in the pre-DBS group (*r* = 0.53; *p* = 0.001) while no significant correlation was found in the DBS group.

## DISCUSSION

The question of the safety of STN-DBS and its effects on cognition and behavior is still open and controversial. To date, while some studies have evaluated the effect of the surgical procedure on mood, apathy, and anxiety, to our knowledge no study has been conducted on alexithymia, despite the fact that this affective dysregulation has a high prevalence – about 21% – in the population of PD patients (not DBS treated) against 13–15 % in the general population (Costa et al., 2010; Assogna et al., 2012).

Recent evidence has reported that PD patients treated with DBS of STN could be at higher risk of suicide with respect to PD patients not treated with DBS (Voon et al., 2008; Strutt et al., 2012), in so much as suicide was defined as “one of the most important potentially preventable risks for mortality following STN-DBS for PD” (Voon et al., 2008). This evidence highlights the need for further studies on the neuropsychiatric effect of DBS surgery, especially in the matter of depression and emotional dysregulation disturbances, since these factors can be predictive of suicidal ideation and/or suicidal attempts (Voon et al., 2008). Indeed, completed suicide has been found to be associated with post-operative depression (Voon et al., 2008). In addition, suicidal ideation seems to be more common in alexithymic subjects than non-alexithymic subjects (Hintikka et al., 2004). One study carried out on the general population has suggested that when depression presents alexithymic features, the affected person has an additive impact on the risk of suicidal ideation (Hintikka et al., 2004).

The principal aim of this study was to investigate whether STN-DBS would affect alexithymic features, by means of a comparison of a matched control group of PD patients under pharmacological treatment only and a matched group of healthy subjects. Our results suggest that STN-DBS does not affect alexithymia. The TAS score results (total score and three factors) highlighted no significant differences between surgically and non-surgically treated PD patients. In actual fact, we found similar percentages of alexithymia in the DBS and pre-DBS groups: 29.6% (8/27) and 31.6 (12/38), respectively.

This said, the DBS and pre-DBS groups both showed twice the prevalence of alexithymia compared with the C group: 14.8% (4/27). As far as the three features of alexithymia were concerned, the results evidenced a significant difference only in the factor concerning “difficulty in distinguishing physiological from emotional state” (F1). In this case, too, as well as for the TAS total score, the DBS and pre-DBS groups both evidenced a statistically significant higher score than the subjects of the control group.

Irrespective of the effect of DBS, our results confirmed the high prevalence of alexithymia in medically treated PD patients (Costa et al., 2010; Assogna et al., 2012). Our prevalence was even higher than that of Costa et al. (2010) and Assogna et al. (2012) 22 and 21%, respectively, against 31.6% in our sample.

This difference could probably result from differences in patients’ characteristics, such as severity of disease and dopaminergic treatment dosage, both higher in our sample (see **Table 1**) than in these other studies sample (Costa et al., 2010; Assogna et al., 2012). As far as the TAS subscales are concerned, we found a significant difference in the “difficulty identifying feelings” (F1) factor between the pre-DBS and C groups. This result agrees with the evidence of Assogna et al. (2012), while another study has shown a difference only in the F2 subscale “difficulty describing feelings” (Costa et al., 2010).

Neuropsychological tests results showed a significantly worse performance of DBS patients (vs. pre-DBS) on some attention/executive tests, especially on the phonemic fluency task. This evidence is in line with previous studies focusing on cognitive STN-DBS outcome (Parsons et al., 2006; Witt et al., 2008; Castelli et al., 2010). In addition, while confirming that DBS-STN did not lead to general cognitive deterioration, neuropsychological results highlighted that specific cognitive domains, such as attention and executive functions, can by negatively affected by this surgical procedure.

In regards to this aspect, it is important to note that alexithymic symptoms can be related to neuroanatomical dysfunction, i.e., right hemisphere or frontal lobe damage, especially anterior cingulate cortex dysfunctioning (Berthoz et al., 2002; Costa et al., 2007; Assogna et al., 2012). Even if this issue is still controversial – a recent study suggest that “the personality trait of alexithymia might be associated with fewer morphological abnormalities than previously assumed” (Heinzel et al., 2012) – it is important to remark that alexithymia can be related and, possibly consequent, to the other symptoms usually present in PD patients, i.e., depression, apathy, and cognitive impairment, all of which may have overlap with alexithymia. So, even if our correlation results did not show a relation between cognitive tests and alexithymia results, we can not exclude that alexithymic features can be related or consequent to cognitive deficits. Further studies on these aspects are needed.

As far as depressive symptoms are concerned, our results evidenced that the pre-DBS group was more depressed than the C group, while no difference emerged between the DBS and C groups. This evidence seems to confirm the results of previous studies showing a positive effect of STN-DBS on depressive symptoms (Castelli et al., 2006), even though, as previously stated, this issue is still debated and other studies have pointed out a post-surgery increase in depressive symptoms (Strutt et al., 2012).

To date, there is no univocal explanation for depressive symptoms amelioration after STN-DBS. The improvement in depressive symptoms could be a consequence of motor symptoms reduction as well as it could reflect the intrinsic role of the STN in non-motor circuits and related symptoms (Witt et al., 2012).

The results for alexithymia and depression suggest that circuits beneath alexithymia and depression are not totally overlapped (Poletti et al., 2011). Although many studies have pointed out a strict correlation between depressive symptoms and alexithymic traits in both the general and clinical population (Hintikka et al., 2004; Costa et al., 2010), a recent study suggests that alexithymia is a depressive-independent phenomenon in PD patients (Assogna et al., 2012). Our results seem to indirectly confirm this last hypothesis: while depressive symptoms are lower after surgery (comparable to C group level), this is not the case with alexithymic features. Moreover we found a significant correlation between alexithymia and depression only in the pre-DBS group but not in the DBS group. On this basis, depression and alexithymia can be considered two distinct but partially overlapped clinical phenomena. Further, it can be supposed that STN-DBS intervention could influence some circuits implicated in depression but not in alexithymia.

In conclusion, the findings of this study suggest that STN-DBS intervention seems to have some effect on depressive symptoms, but not on alexithymic traits. Actually, on the one hand depressive symptoms in DBS patients are comparable to those in the C group, while pre-DBS patients were found to be more depressed than controls subjects. On the other hand alexithymia was present in the same percentage in the two groups of patients (DBS and pre-DBS). Furthermore, the comparison with a control group of healthy matched subjects allows us to confirm the high prevalence of alexithymia in PD patients. This evidence points out once again the importance of taking into account psychological aspects as well as cognitive outcome and the relation between these two domains in order to conduct a multidisciplinary assessment and treatment for patients undergoing STN-DBS surgery.

### LIMITATION

Provided that, to our knowledge, this is the first study investigating alexithymia in STN-DBS treated PD patients and that we used a case-control design with both a matched control group of PD patients not treated with DBS and a matched group of healthy participants, further studies using a prospective design (pre- vs. post-operative evaluation) are needed in order to confirm our findings and to draw more definitive claims.

In addition we could not investigate the possible role of antidepressant medications on psychological variables due to the great number of incomplete or missing psychopharmacological data.

## Conflict of Interest Statement

The authors declare that the research was conducted in the absence of any commercial or financial relationships that could be construed as a potential conflict of interest.
